# MULTIMETHOD COMMUNITY-BASED DEMENTIA CARE EDUCATION TO EMPOWER CARE PARTNERS AND IMPROVE WELLNESS

**DOI:** 10.1093/geroni/igad104.1659

**Published:** 2023-12-21

**Authors:** Rollin Wright, Janice Whitaker, Peg Chabala

**Affiliations:** Penn State Health at Hershey Medical Center, Hershey, Pennsylvania, United States; Nese College of Nursing, Penn State University, State College, Pennsylvania, United States; Penn State University, University Park, Pennsylvania, United States

## Abstract

Inspired by the Medicare Cognitive Assessment and Care Plan Services visit, we describe an Age-Friendly 4Ms curriculum developed to empower informal dementia-care-partners to understand dementia, develop skills to communicate and work with people living with dementia (PLWD), and plan for the future with dementia. Curriculum was developed by an interdisciplinary team and included the integration of evidence-based approaches to care, such as the Positive Approach® to Care model. Through 4 interactive workshops (Understanding Dementia-related Brain Changes, Understanding Behaviors as a Form of Communication, Resources for Dementia Caregivers, Planning for the Future with Dementia) we introduced and had care partners process and practice material to help them navigate everyday challenges faced by PLWD with the aim of assisting them to find opportunities to help improve quality of life with the disease for both themselves and their PLWD. We reached a total of 146 attendees in 3 rural and suburban communities in Central Pennsylvania. Fifty-nine (40.4%) people attended 1 session, 39 (26.7%) people attended 2 sessions, 22 (15.1%) people attended 3 sessions, and 26 (17.8%) people attended all 4 sessions. Across all four sessions, more than 80% of attendees reported knowledge and skill gains, and most attendees reported meeting session objectives. Final evaluation results suggest attendees wanted a way to stay informed, stay connected, and remain supported by the program.

